# Supplementary material for: Evidence from Buhais Rockshelter for human settlement in Arabia between 60,000 and 16,000 years ago
Source: Nat Commun. 2026 Mar 23;17:2502. doi: 10.1038/s41467-026-70681-z (PMC13009211; doi:10.1038/s41467-026-70681-z)
Supplement: Supplementary file 1 — Supplementary Information [file 41467_2026_70681_MOESM1_ESM.pdf]

Supplementary information for

Evidence from Buhais Rockshelter for human settlement in Arabia between 60,000 and 16,000 years ago

K. Bretzke, S. Kim, S. Jasim, E. Yousif, F. Preusser, G.W. Preston, F. Pallottino, A. G. Parker

Supplementary Note 1: Site information

Sharjah Archaeology Authority mapped the site as Buhais 84. In the late 1990s Sharjah Archaeology Authority excavated the Iron Age burials at the site. The 1.7 m deep Pleistocene sediments were not excavated at that time. Test excavations in 2017 revealed the potential for Paleolithic records through recovering two archaeological layer named archaeological horizon (AH) I and II. The extension of the excavated area in 2019 led us to rename the AHs defined 2017. This was due to the removal of one of the big rock fall blocks during the extension of the excavated area in 2019. Under this block there was sediment preserved that was stratigraphically above AH I from 2017. We decided to name the upper-most layer AH I and renamed previous layer AH I to AH Ia. This allowed us to keep AH II as it was defined in 2017. In five years of excavation at the site, we identified seven distinct geological layers, with four of them containing archaeological material (Supplementary Figs. 1-7).

The lowest layer, GH 3, provides contact to the bedrock. The layer is about 30 cm thick with very-well visible contact to layer GH 2 above. This sandy layer is matrix supported and contains about 5% clasts. The matrix consists of well-sorted medium to fine sand, brown in color (Munsell 7.5 YR 5/4). GH 3 contains the oldest archaeological layer AH III (Table 6). OSL dating of two samples yield a mean age of 124 ka ± 6 ka (Buhais 3: 123 ± 10 ka; Buhais 7: 125 ± 9 ka). Sediments immediately above AH III, devoid of archaeological material, were dated to 95 ka ± 9 ka (Buhais 6).

GH 2 is a 25-30 cm thick gravel rich layer with well visible contact to GH 1c, GH 1d and GH 3 (Supplementary Fig. 1, and Fig. 4 of the main text). The layer is almost clast supported with about 60% limestone clasts. Clasts range in size between 15 and 50 cm. Shapes are dominated by flat variants with rounded edges. The layer contains a relatively high proportion of chert fragments, both natural and artificial. Limestone clasts are embedded in a very poorly sorted matrix composed of small pebbles to medium sand. The reddish-yellow layer (Munsell color 5 YR 7/6) is relatively loose, almost unconsolidated. GH 2 contains AH II. OSL dating of one sample from AH II yielded an age of 59 ± 5 ka (Buhais 4).

Stratigraphically the next younger layer is GH 1d, which occurs exclusively in the western profile as an archaeologically sterile local feature of about 15 cm thickness. This feature thins out towards the south at about Y=38 m (Supplementary Fig. 1). GH 1d is characterized by an increased sand fraction and features well-established contacts to GH 1c and GH 2. The layer is matrix supported, only lightly consolidated and consist of well sorted medium to fine sand with only about 10% clasts.

GH 1c is a 25-30 cm thick layer with blurred contact to layer GH 1b above, but well visible contact to GH 2 and 1d below. GH 1c terminates at the bedrock step at about x = 29 m (see main text Fig. 4). The layer is matrix supported with about 50% limestone clasts 5-8 cm in size. Clasts are flat with rounded edges. Few globular rocks, 10-12 cm in size, occur. GH 1c is weakly consolidated and consists of poorly sorted very coarse to medium sand with some pebbles. The layer appears yellowish red in color (Munsell 5 YR 5/6). Very few artifacts occur in GH 1c, mainly in the south-western part of the excavation. Layers GH 1d and GH 1c form together a well-established border between GH 2 and GH 1b, which provides a clear separation of the site's Middle Palaeolithic (MP) and Upper Palaeolithic (UP) layers.

The next younger layer in the sequence is GH 1b, which is about 60 cm thick in the eastern part of the excavation and thins to about 15 cm in the West. Significantly increased consolidation of this layer let borders to GH 1a and 1c become easy to identify. GH 1b is matrix supported with about 50% limestone clasts ranging in size between 10 and 20 mm. Many clasts are flat and sub-angular, but we observed an increase in the proportion of rounded clasts. GH 1b is well consolidated, but poorly sorted. The matrix is composed of small gravel to medium sand and light reddish-brown in color (Munsell 5 YR 6/3). There are many larger limestone rocks, about 5 to 15 cm in size. GH 1b extends over the step in the bedrock, which is not parallel to the backwall of the modern rock shelter, suggesting a change in the orientation of the opening. A thin layer of artifacts was identified within GH 1b. One OSL sample (Buhais 8) has been collected from this layer, which provides an age estimate of 35 ± 3 ka.

Layer GH 1a has built up behind a larger rock fall block, which limits the layer's spatial distribution to the eastern part towards the modern backwall of the rock shelter (see Fig. 4 in the main text). There is only blurred contact to GH 1. Due to increased consolidation in GH 1b, contact between GH 1a and 1b can be identified relatively well. GH 1a is ca. 35-40 cm thick and thins out towards the West. The layer stops behind a large rock fall block at ca. x= 28.5 m. GH 1a is matrix supported with a clast proportion of about 20%. Clast size ranges from c. 5-12 mm. Clasts are mainly flat angular limestone fragments. Matrix consists of poorly sorted very

coarse to medium sand. Very few larger limestone clasts ranging in size between 5 and 10 cm occur. GH 1a is brown in color (Munsell color 7.5 YR 4/3), moderately consolidated. OSL dating of two samples from GH 1a indicates a mean depositional age of 35 ka ± 5 ka (Buhais 1: 30 ± 2 ka, Buhais 9: 41 ± 2 ka). GH 1a contains the lithic assemblage of AH Ia. Given indistinguishable ages for GH 1b and GH 1a, we combine the few finds from GH 1b with those from GH 1a into one AH Ia.

GH 1 is the top layer of the Buhais Rockshelter Pleistocene sequence, which has partly been exposed during the Iron Age excavations in the 1990s. The central part of the Paleolithic excavation was initially covered by big limestone blocks, representing a phase of overhang collapse. We removed these blocks and recovered immediately underneath lithic artifacts of AH I. Layer GH 1 is about 40 cm in thickness, with an increase in the western part to about 50 cm. The lower border to GH 1a is blurred since this layer is largely matrix supported with about 10% of small, 2-5 cm long, limestone clasts, which often lay flat and feature sharp edges. Only few rocks up to 20 cm in size occur in this layer. Most of them feature rounded edges. The matrix of GH 1 consists of poorly sorted coarse to fine sand, which is yellowish-brown in color (Munsell color 10YR 5/4). GH 1 is relatively dry and loose and contains AH I. OSL dating of two samples from within the layer indicates a mean depositional age of 16 ka ± 2 ka (Buhais 5: 14 ka ± 1 ka, Buhais 10: 17 ka ± 2 ka).

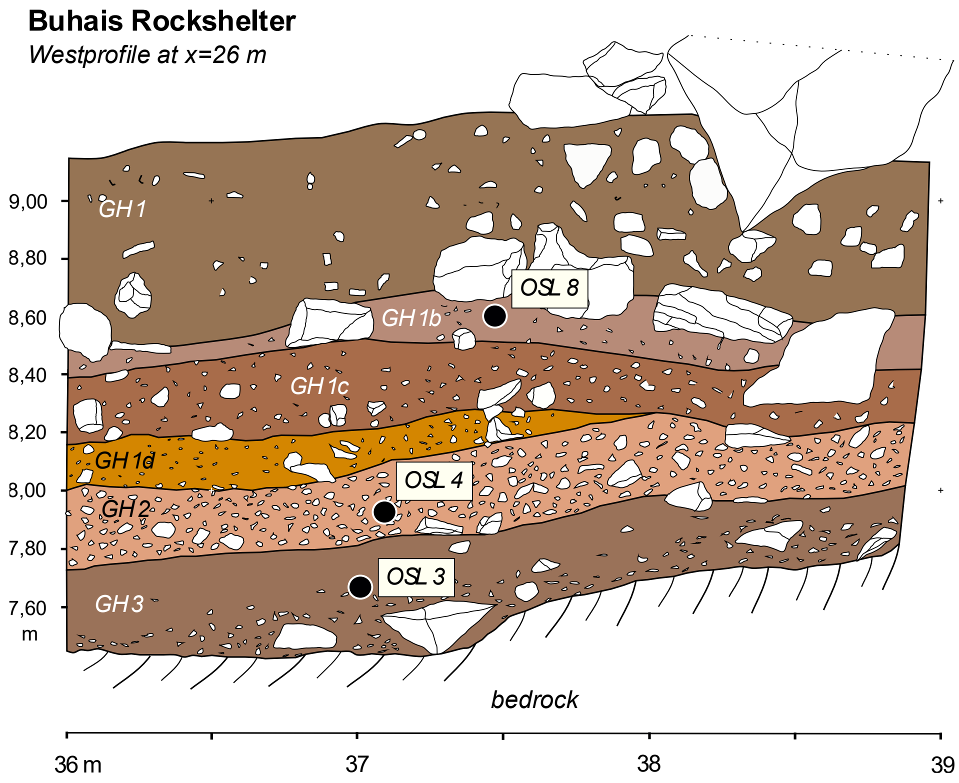

Supplementary Figure 1. Drawing of the western profile showing the geological layers (GH) and the location of OSL samples taken from this profile.

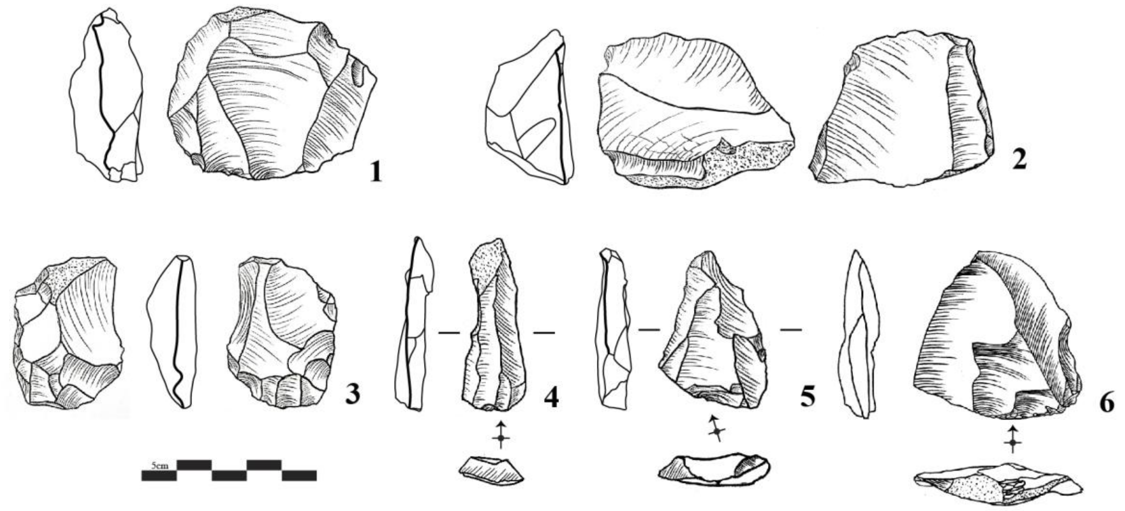

Supplementary Figure 2. Examples of lithic artifacts from AH III. 1: preferential Levallois core with bidirectional preparation, 2: bifacial reduction core, 3: preform bifacial piece, 4: blade, 5-6: triangular non-Levallois flakes. (Drawings SK).

Supplementary Table 1. Basic characteristics of lithic assemblages from Buhais Rockshelter.

**Buhais Rockshelter**	**AH I**		**AH Ia**		**AH II**		**AH III**	
	**n**	**%**	**N**	**%**	**N**	**%**	**N**	**%**
Flakes	625	50,9	262	49,9	719	69,2	132	62,0
Blades	260	21,2	143	27,2	97	9,3	19	8,9
Bladelets	149	12,1	31	5,9	13	1,3	0	0,0
Cores	36	2,9	21	4,0	58	5,6	15	7,0
Angular Debris	159	12,9	68	13,0	152	14,6	47	22,1
**Total**	**1229**	**100**	**525**	**100**	**1039**	**100**	**213**	**100**

Supplementary Table 2. Number of tool types documented in the different layers. BB: Backed Blade/Bladelet, Bo: Borer, Bu: Burin, E: Endscraper, M: Microlith, SR: Simple Retouch, T: Truncation, N-D. Notch-Denticulate, S: Sidescraper, P: Point. The biface mentioned in the main text is counted here as point.

**AH**	**BB**	**Bo**	**Bu**	**E**	**M**	**SR**	**T**	**N-D**	**S**	**P**	**Total**
I	12	7	17	30	4	35	4	5	5	20	139
Ia	0	0	13	34	0	23	6	3	3	12	94
II	0	0	1	5	0	1	0	25	4	4	40
III	0	0	0	0	0	0	0	5	2	0	7

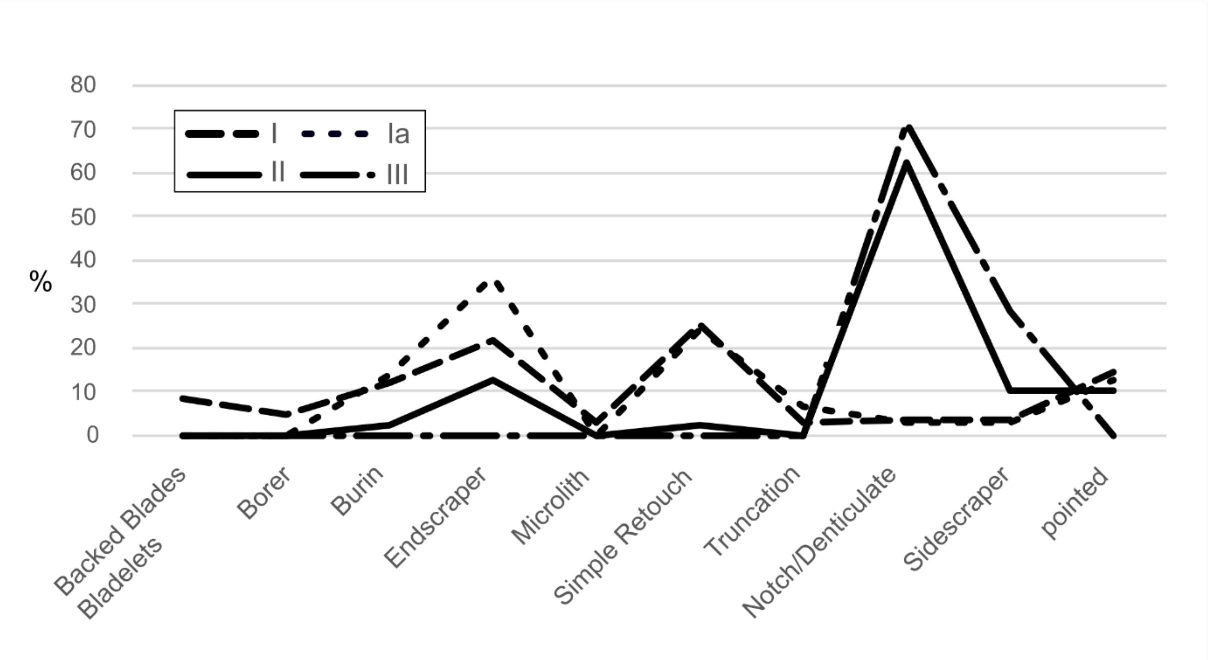

Supplementary Figure 3. Frequencies of different tool types in archaeological layers AH I, Ia, II and III. Please note the marked shift from predominant notches and denticulates in AHs II and III to a preference for endscrapers and burins in AHs I and Ia. For counts please see table 2.

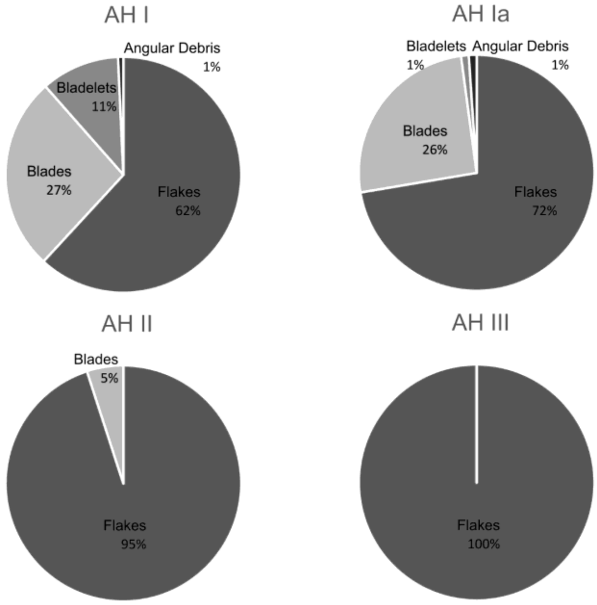

Supplementary Figure 4. Frequencies of tool blank types per archaeological layer (AH). Please note the increased frequency of blade and bladelet blanks in the UP layers AHs I and Ia (upper row) compared to almost exclusively flakes being used for tool production in the MP layers AHs II and III (lower row).

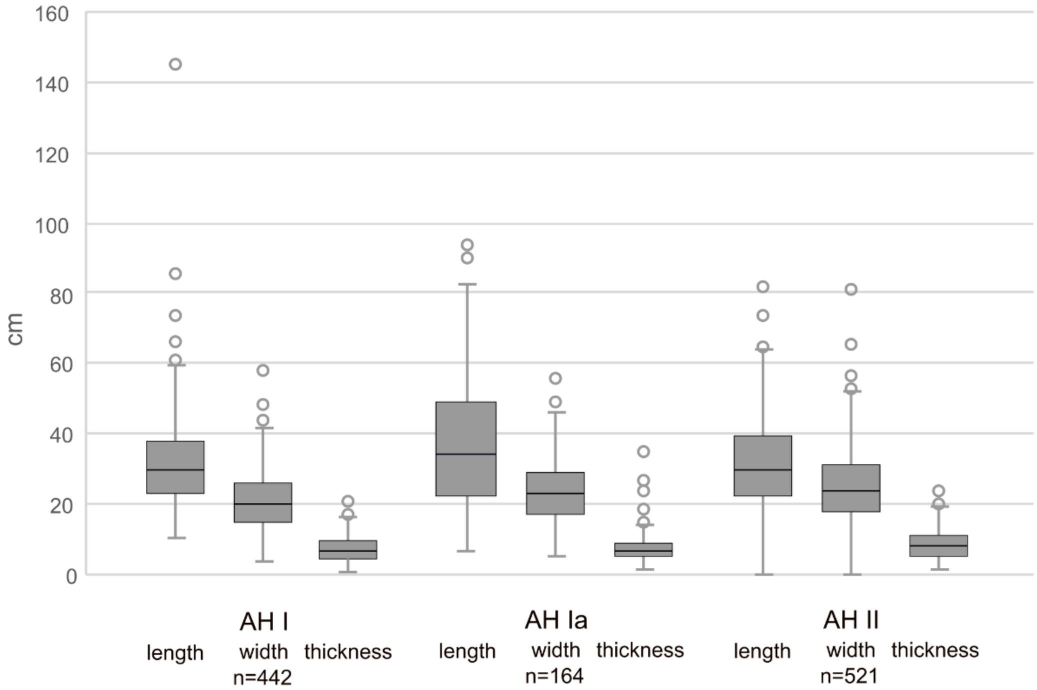

Supplementary Figure 5. Length, width, thickness values of AHs I, Ia, and II shown as boxplots. This graph shows only results from measurements of complete pieces. The n shown below each layer indicates the number of artifacts considered here. Please note that artifacts get narrower and thinner in the UP layers compared the assemblage from the LMP layer in AH II. Please further note that the horizontal line represents the median; box limits represent first and third quartile, whiskers show maximum and minimum, circles represent outliers that are larger or smaller the 1.5x interquartile range.

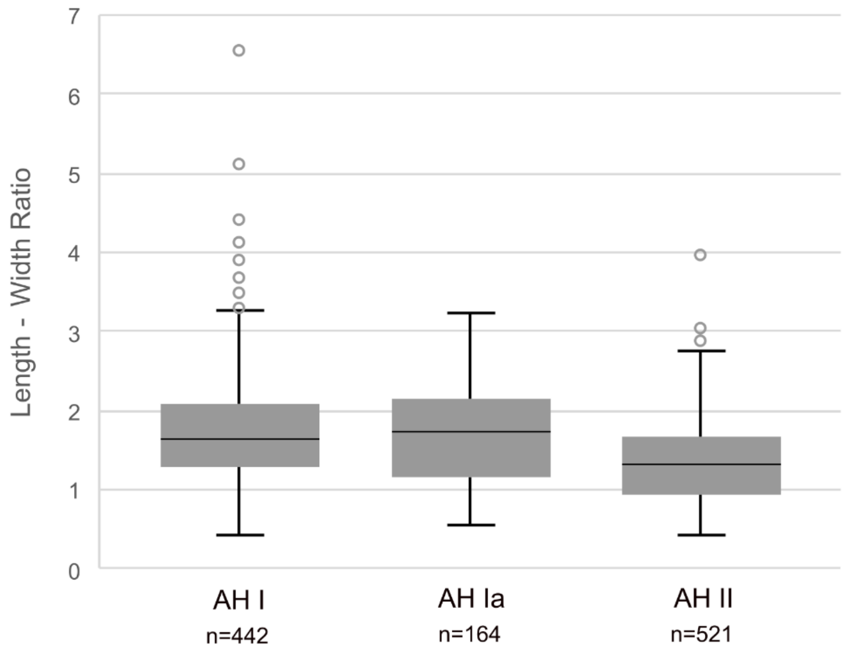

Supplementary Figure 6. Length to width ratio of complete artifacts in the two UP layers (AH I and Ia) and the LMP layer (AH II). Please note that there is a marked increase in elongation from the LMP in AH II to the UP in AHs I and Ia. This observation is linked to the systematic blade and bladelet production recorded in AHs Ia and I. Please refer to Supplementary Fig. 5 for the characteristics of the box plots.

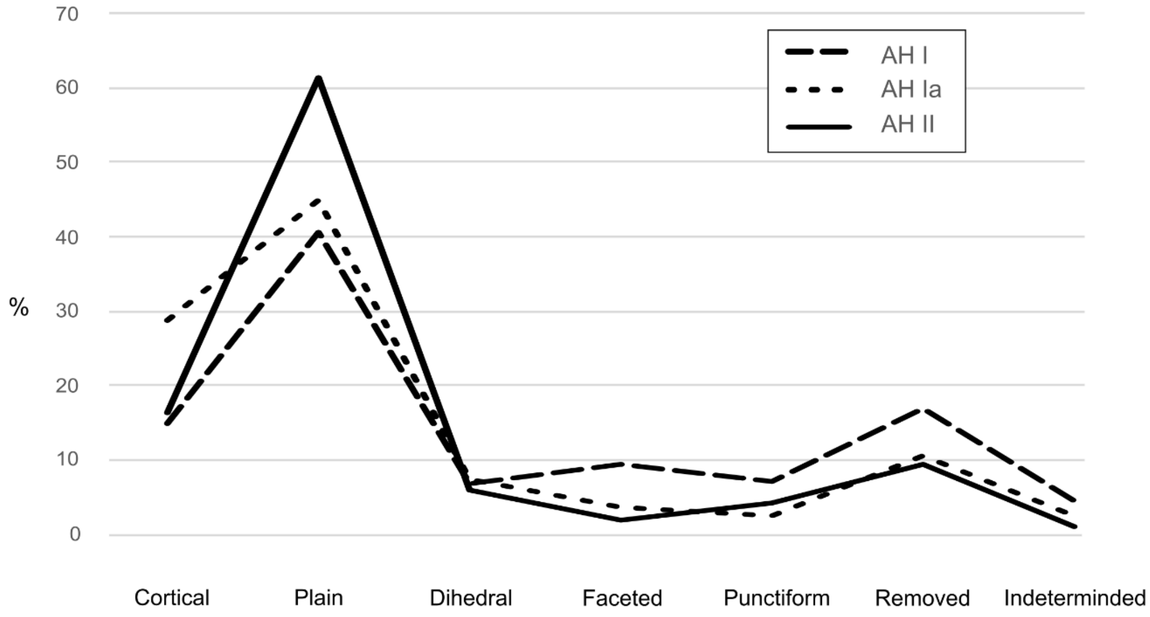

Supplementary Figure 7. Frequency of different types of striking platform recorded in AHs I, Ia and II.

Supplementary Note 2: Palaeoenvironmental information

Facies analysis was applied to the 4.7 m deep P6 sequence, to determine fluvial and aeolian depositional architectures, and to provide insights palaeoenvironmental conditions during their deposition. Two key facies associations were identified with (1) fluvial channel sediments comprising sands and gravels, and (2) laterally extensive aeolian deposits, overlying fluvial deposits, associated with periods of relatively more arid conditions at a regional scale. Eight distinct facies were identified (Table 3). Supplementary Figure 8 shows the detailed physical and geochemical analyses along with facies referred to in the main text.

Supplementary Table 3. Sedimentary facies observed in the P6 section.

No	Facies Code	Facies	Sedimentary Structures	Interpretation
1	Gcm	Clast supported massive gravel	Ophiolite Gravel, medium coarse gravel with pebbles and occasional small cobbles, cemented	Unidirectional flow in higher discharges or channel lag deposits. Channel and interchannel areas.
.2	Gcp	Clast supported gravel, planar cross-stratified gravel	Planar cross-beds	Transverse bedforms, migration of gravel bars in a unidirectional high flow regime. Channel area.
3	Sl	Sand, very fine to coarse, may be pebbly	Low-angle (< 15°) cross-beds	Scour fills, humpback or washed-out dunes, antidunes. Channel area.
4	Sm	Sand, fine to coarse. Massive	Massive, or faint lamination	Sediment-gravity flow deposits. Rapid deposition of hyper-concentrated flow, unidirectional flow in higher discharges. Channel area.
5	Ss	Sand, fine to very coarse, may be pebbly	Broad, shallow scours	Scour fill. Channel area.
6	Fm	Silt, mud, Massive.	Massive due to flocculation or intense bioturbation.	Deposition of sediments from suspension. Channel and interchannel areas
7	Fl	Sand, silt, mud, which might present bioturbation.	Fine laminated sand, silt and mud, very small ripples	Deposition of suspended load by settling out in standing water. Channel and interchannel areas.
8	Sem	Fine to medium sand, moderately- to well-sorted, sub- to well-rounded grains> massive. Occasionally consist of millimetrically spaced inverse graded lamination, and pin-stripe lamination.	Massive, some minor bedding structures.	Aeolian dune sand (AD)

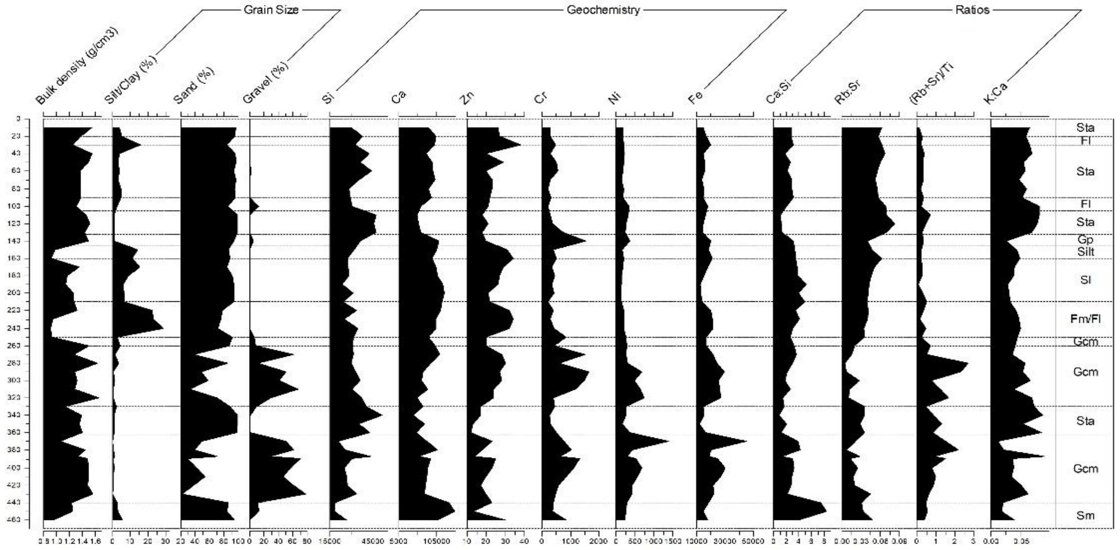

Supplementary Figure 8. Physical, geochemical (elemental and ratio) and facies from the Mleha P6 section.

Supplementary Table 4. Sediment description Huwaimi R1, Ar Rashidiyya.

Depth (cm)	Description	Facies
0-36 cm	Finely banded sands, alternating between orange medium sands (10YR 6/4) and white fine sands (2.5Y 6/3 6/4). Roots. GUAQ14_HMW_OSL 1 @ 26 cm.	Sem
36-46 cm	Cemented silts and very fine sands, very fine wavy banding. 2.5Y 6/3. Sediment-filled animal burrow. Sharp lower boundary.	Fl
46-66 cm	Very compacted clays (darker) and silts (lighter). Much darker in colour than above units. Sharp lower boundary. 3 colours identified 2.5Y 7/3 (lightest), 10YR 6/3 (medium), 2.5Y 4/3 (darker).	Fl
66-93 cm	Homogenous fine sandy silts. 10YR 6/3	Fm
93-158 cm	Very compacted clays (darker) and silts (lighter). Similar to unit 3 with larger banding layers. Sharp lower boundary. 3 colours identified 2.5Y 7/3 (lightest), 10YR 6/3 (darker), 2.5Y 4/3 (organic).	Fl
158-170 cm	Compacted medium sands, homogeneous unit. 10YYR 6/4. GUAQ14_HMW_OSL 2 @ 160 cm.	Sem

The Huweimi sequence comprises 6 units. The basal sediments (170-158 cm) comprise compacted, homogenous medium aeolian sands (Facies 8, Sem) and represent dune sands that form the base of the interdune. These are overlain by finer-grained banded silts and clays between (158-36 cm) which represent the main phase of lacustrine deposition (Facies7, Fl). Within the fine-grained silts, a slightly coarser sandy silt unit occurred at 93-66 cm (Facies 8, Fm). The top of the sequence (36 cm to 0 cm, Facies 8, Sem) comprise orange medium sands and are aeolian in origin. 40 samples were collected for sedimentological and geochemical

analyses. Detailed physical and geochemical results mentioned in the main text are shown in Supplementary Figure 9. Two OSL dates were measured from 160 cm and 26 cm and to bracket the phases of lacustrine sediment deposition and are shown in Table 5.

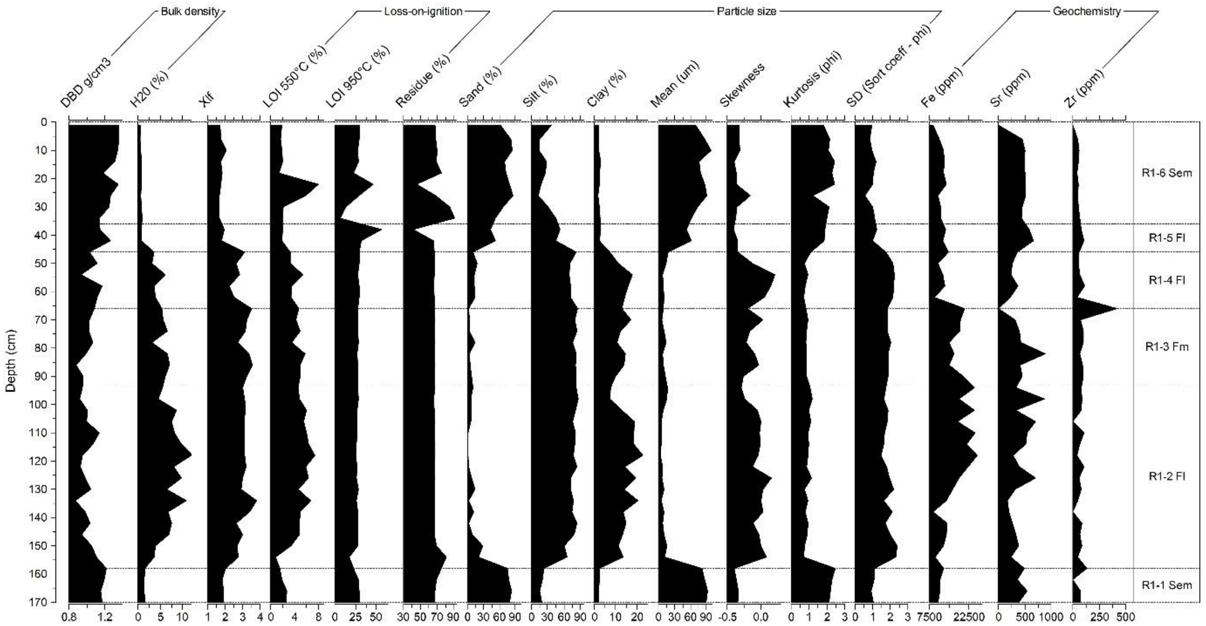

Supplementary Figure 9. Physical, and geochemical (elemental) analyses from the Huweimi (R1) section.

Supplementary Note 3: Age determination

Sample preparation and De measurements

At the OSL dating lab of the University of Freiburg, the quartz fraction was extracted from the material collected in opaque bags by sieving, chemical treatments (10% HCl), density separation (liquids with ρ = 2.70 and 2.58 g cm-3), and HF etching (40% for 60 min, followed by 10% HCl treatment >60 min; no re-sieving). Equivalent dose (D_e) was determined using 1 mm aliquots (ca. 50 grains). Considering that only about 3% of all grains in the region produce acceptable luminescence characteristics[1] results in an average number of about 1.5 grains per aliquot, which will dominate the signal. Hence, the small aliquot approach used here will be very close to a single-grain level.

All measurements were done on a Freiberg Instruments Smart device[2] using blue light stimulation (458 nm at 50 mW cm^{-2}) and the combination of a Hoya u-340 and Delta BP 365/50 interference filter. The OSL decay curves are quite bright and dominated by the fast component, (Supplementary Fig. 10 A-C). For example, a typical aliquot of samples BUHAIS1 emits 7000 photon counts during the first 0.4 s of stimulation for a regenerative dose of ca. 20 Gy (background ca. 80 cts/0.4 s). The single aliquot regenerative dose (SAR) protocol was used[3] and preheating at 230°C for 10 s was identified in dose recovery test. This was done by bleaching the sample (BUHAIS 1) in the OSL reader (3x 60 s blue stimulation as above), apply a test dose of ca. 20 Gy, and run the full SAR protocol as used for D_e determination. We gained a dose recovery ratio of 1.02 ± 0.03 (n = 24). IR stimulation in the SAR cycle was used to check for potential feldspar contamination but to not reveal any problem. About 40-50 aliquots were measured for the rock shelter and some of the section samples, when displaying very wide D_e distributions. For three samples, the amount of quartz was so low that only 11 replicate measurements were possible but these are Holocene samples given here only for completeness.

Dose response curves were fitted in Analyst 4.57 using either a single-exponential (for D_e values < ca. 50 Gy; Supplementary Fig. 10A and B) or a double-exponential saturating function (for D_e values > ca. 50 Gy; Supplementary Fig. 5C), including an instrumental uncertainty of 2%. Of the total number of measured aliquots, a small number had to be rejected when failing the rejection criteria of Wintle and Murray[4], i.e. recycling ±10% of unity within uncertainties, recuperation less than 5% of the natural signal, test dose signal >3x background. The number of measured and totally accepted aliquots is given in Table 6.

D_e analyses and determination of average dose absorbed

The D_e distributions of all samples are given as Abanico plots (Supplementary Fig. 11; cf.[5]) and average D_e was calculated using the Central Age Model (CAM), the Minimum Age Model (MAM), and for sample BUHAIS3 the Finite Mixture Model (FMM; cf.[6]) instead of MAM. For the FMM, three components were assumed, and average values were computed using sigma_b values between 0.04 and 0.26. The value showing the best fitting criteria (BIC) was used for further calculations. The reasoning behind using the FMM is justified by the assumption that three different processes were being involved for placing grains at the position encountered during sampling. The main source of input expected is aeolian influx, which will deposit grains that will likely be well-bleached, as shown by Bretzke et al.[7] for modern analogues from the nearby Jebel Faya site. A second source of grain input will be the delivery of grains from weathering of the rockface, as already discussed by Armitage et al.[1] for Jebel Faya. While the amount of quartz in the limestone is quite low, the volume of roof material that disintegrated with time is quite substantial. The OSL signal in such grains will likely not have been zeroed at the time of deposition due to the short transport distance and will produce a population of D_e values that are not related to the age of sediment deposition. Such D_e values must not be considered when calculating the average dose absorbed since burial. The third input of grains might be by post-depositional mixing (i.e., bioturbation) that will introduce grains with an OSL signal that reflect a daylight exposure time younger than the formation of the archaeological layer (cf. [1]). As only sample BUHAIS3 shows an apparent distribution at the lower edge, the latter process seems to play a minor role for the samples under consideration.

Almost all samples show positively skewed D_e distributions with overdispersion values (OD) >0.20, which are typical for samples consisting of a mixture of well- and poorly-bleached grains. This calls for the application of the MAM. A key value for this is the input parameter sigma_b, which represents the expected spread of values without the effect of partial bleaching. For the rock shelter, sample BUHAIS7 shows the lowest od (0.17) but still a positive skewness, which let us to use a sigma_b value of 0.10 for the MAM calculation. Manually removing D_e values until it reaches od = 0.10 indeed results in a normal distribution of D_e values, which gives confidence in the applied input value. This value is very close to the value observed for one sample from the Huweimi (0.11) but the second sample showing even a lower value (0.05). Interestingly, for section P6 three samples show normal (Gaussian) distributions with od values of 0.20 (2x) and 0.17. These appear to represent well-bleached samples and have the same od as reported by Mueller et al.[8]. Hence, for section P6 a sigma_b value of 0.20 was used for MAM calculations. The discrepancy observed between sides remains unclear.

Dose rate determination and calculation

Material for high-resolution gamma-spectrometry was dried, grinded, and measured at VKTA Rossendorf e.V. for the activity of K, Th and U (cf.[9]). We regard this approach as most appropriate for the samples under consideration for three reasons. 1) The method allows determining the activity of different isotopes from the Uranium decay chain and investigating the samples for the presence of radioactive disequilibrium. 2) The method uses rather large amounts of material (400 g), which is expected to compensate for sample inhomogeneity as observed at the nearby side of Jebel Faya[1]. 3) In contrast to in-situ gamma spectrometry, it is not needed to create a hole in the sediment exposure which will require remove material from the area of interest (i.e. the sample taken for De determination). The comparison of the activities determined for U-238 and Ra-226 reveals no indication for the presence of radioactive disequilibrium in any of the samples investigated. Water content was assumed at 3±3% for the blocky material from Buhais Rockshelter, and 4±4 for the sandy deposits but assuming slightly higher values for the lower part of section P6 when approach the phreatic zone (6±2 for P6/2023-2 and P6/2023-2, 6±2 for P6/2023-1). Dose rates and ages were calculated using ADELEv2017 (*76*; add-ideas.de), taking into account longitude (55.5°E), latitude (25.0°N), altitude (178 m), and sample depth below surface (Table 5) for cosmic dose rate (*77*). Considering the nature of the rock shelter and the situation of the excavation (Fig. 2C of the main text), the effect of shield by bedrock is considered negligible. The dosimetric data is compiled in Table 5, the resulting ages are presented in Table 6.

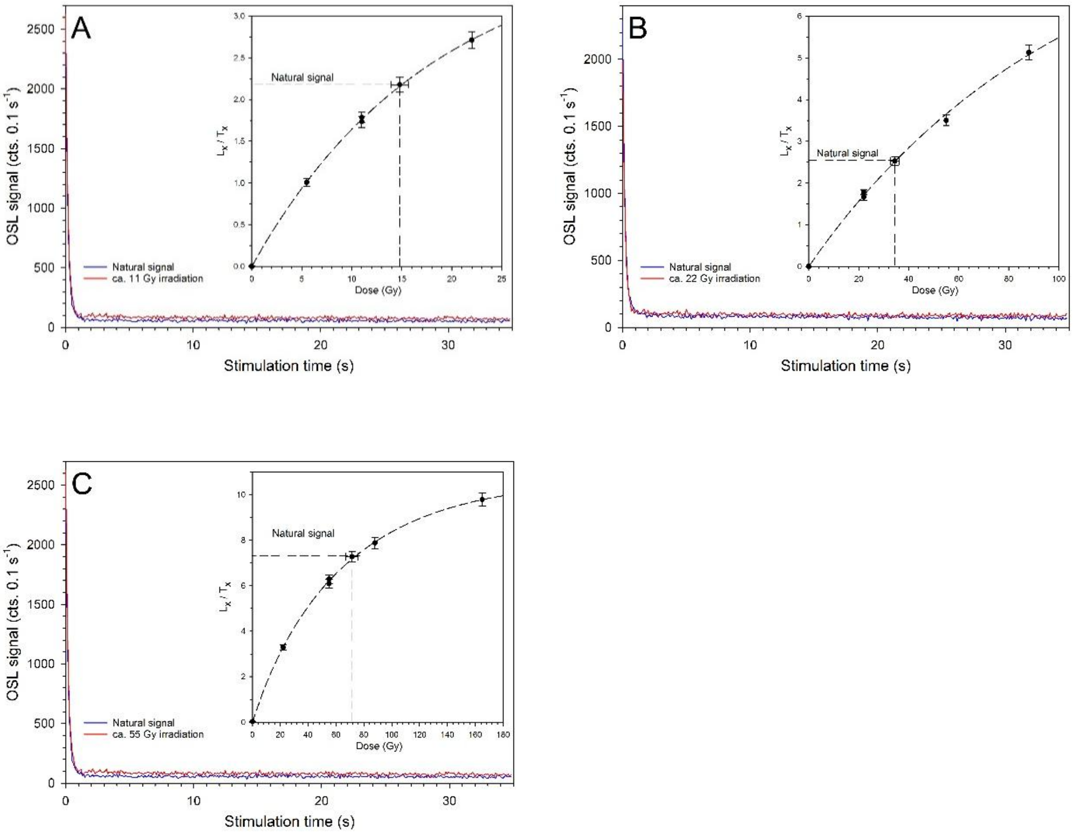

Supplementary Figure 10. Example OSL decay and dose response curves for three samples from Buhais Rockshelter (A: BUHAIS-5, 14.8±1.1 Gy, B: BUHAIS-1, 34.5±1.5 Gy, C: BUHAIS-3, 75.5±5.6 Gy).

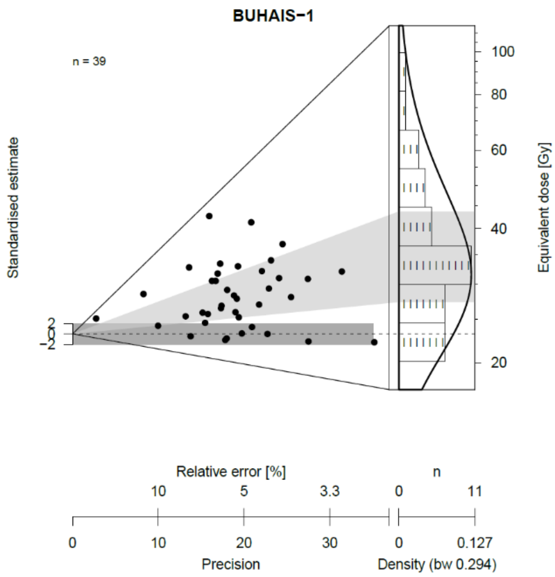
BUHAIS−1
n = 39
Standardised estimate
Equivalent dose [Gy]
100 80 60 40 20
2 0 −2
Relative error [%]
10 5 3.3
Precision
0 10 20 30
n
0 11
Density (bw 0.294)
0 0.127

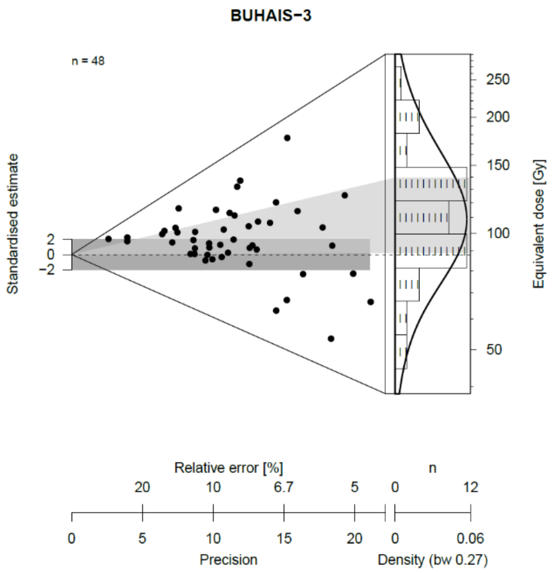
BUHAIS−3
n = 48
Standardised estimate
Equivalent dose [Gy]
250 200 150 100 50
2 0 −2
Relative error [%]
20 10 6.7 5
Precision
0 5 10 15 20
n
0 12
Density (bw 0.27)
0 0.06

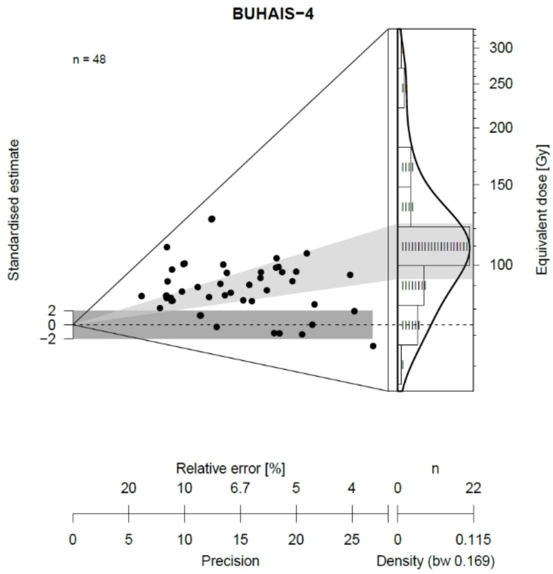
BUHAIS−4
n = 48
Standardised estimate
Equivalent dose [Gy]
300 250 200 150 100
2 0 −2
Relative error [%]
20 10 6.7 5 4
Precision
0 5 10 15 20 25
n
0 22
Density (bw 0.169)
0 0.115

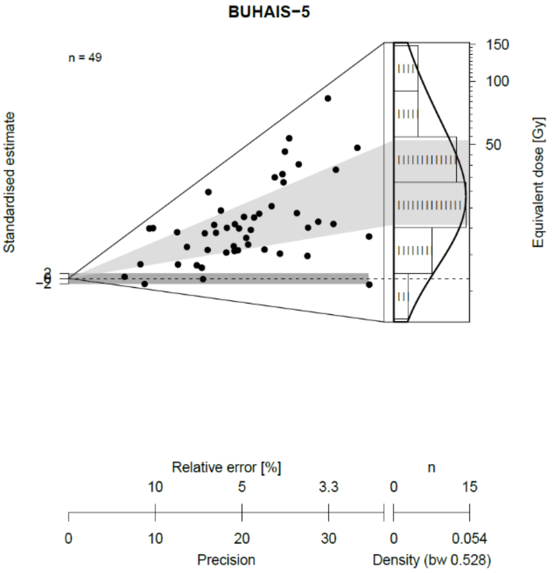
BUHAIS−5
n = 49
Standardised estimate
Equivalent dose [Gy]
150 100 50
2 0 −2
Relative error [%]
10 5 3.3
Precision
0 10 20 30
n
0 15
Density (bw 0.528)
0 0.054

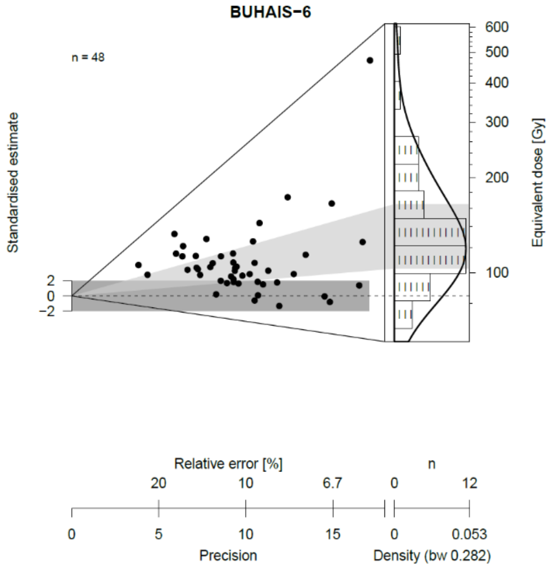
BUHAIS−6
n = 48
Standardised estimate
Equivalent dose [Gy]
600 500 400 300 200 100
2 0 −2
Relative error [%]
20 10 6.7
Precision
0 5 10 15
n
0 12
Density (bw 0.282)
0 0.053

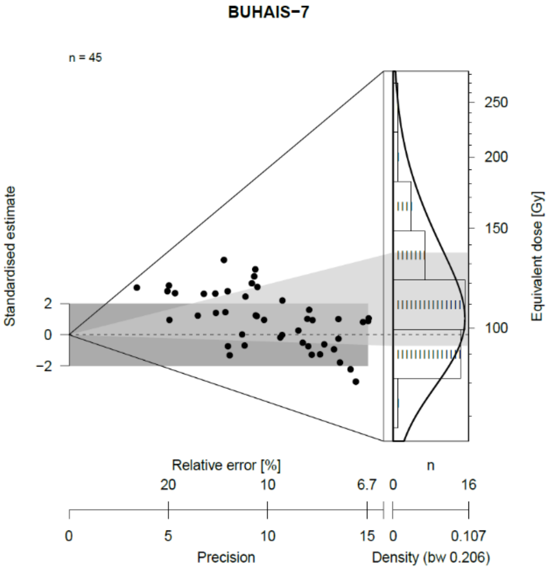
BUHAIS−7
n = 45
Standardised estimate
Equivalent dose [Gy]
250 200 150 100
2 0 −2
Relative error [%]
20 10 6.7
Precision
0 5 10 15
n
0 16
Density (bw 0.206)
0 0.107

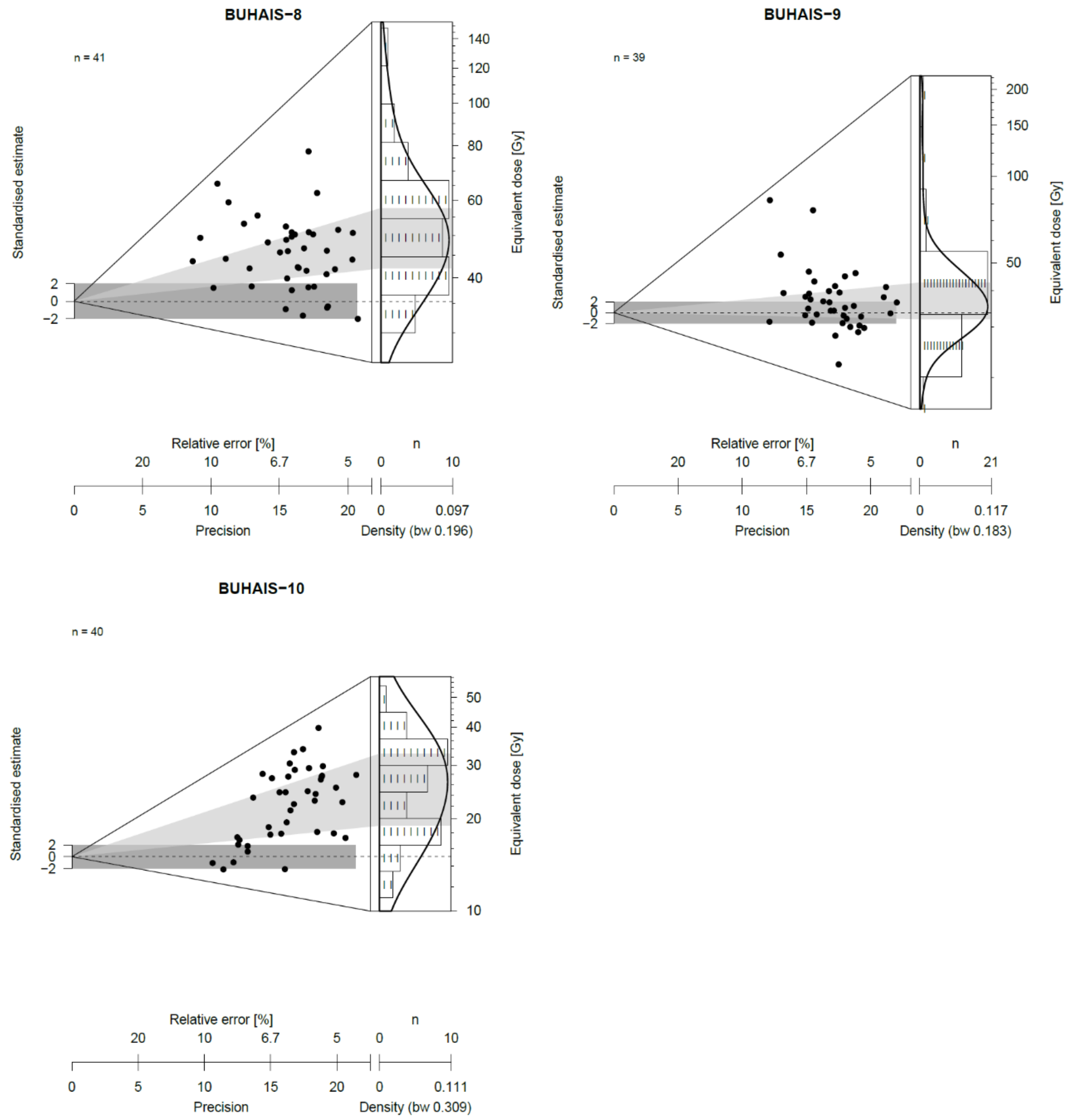

Supplementary Figure 11. Abanico plots[5] showing the De value distributions of the nine OSL samples investigated here (plotted using the R script by Kreutzer et al.[10]). The light grey shaded zone indicates the arithmetic mean value, whereas the dark grey zone highlights the part of the data set included in the age model. The dashed line indicates the average value used for age determination.

Supplementary Table 5. OSL dosimetry data with assigned archaeological horizon (AH); sampling depth below surface; grain size used for D_e calculation; activity of dose-rate relevant elements; resulting dose rate (D).

Sample	AH	Depth (cm)	Grain size (µm)	K (Bq/kg)	Th (Bq/kg)	U [U-238] (Bq/kg)	U [Ra-226] (Bq/kg)	D (Gy ka^{-1})
BUHAIS-1	Ia	65	150-200	108±11	3.8±0.3	11.4±1.6	11.7±0.7	0.78±0.09
BUHAIS-3	III	146	90-160	36±4	1.2±0.1	18.6±2.1	22.4±1.4	0.72±0.05
BUHAIS-4	II	132	90-160	205±22	5.5±0.5	21.8±2.5	19.6±1.3	1.24±0.12
BUHAIS-5	I	18	90-160	109±8	4.0±0.3	8.8±1.4	10.3±0.7	0.82±0.09
BUHAIS-6	above III	122	100-200	130±10	4.0±0.2	14.7±1.8	14.2±1.0	0.89±0.09
BUHAIS-7	III	130	100-200	105±8	3.6±0.2	11.6±1.5	12.6±0.8	0.78±0.06
BUHAIS-8	Ia	69	100-200	150±11	4.8±0.3	15.6±1.8	15.9±1.0	1.00±0.10
BUHAIS-9	Ia	63	100-200	118±9	3.5±0.2	11.8±1.3	10.7±0.7	0.82±0.06
BUHAIS-10	I	22	100-200	123±9	4.3±0.2	11.1±1.3	11.2±0.7	0.86±0.09
HWM1		26	100-150	243±20	9.4±0.6	18.2±2.4	17.3±1.1	1.46±0.06
HWM2		160	100-150	245±21	9.7±0.7	19.0±3	17.0±2.2	1.43±0.06
P6-1		50	100-160	242±4	7.0±0.5	7.7±2.6	11.6±0.2	1.29±0.09
P6-2		70	100-160	251±3	10.8±0.3	13.4±4.4	15.8±0.7	1.46±0.10
P6-3		105	100-160	230±4	6.5±0.4	7.1±2.7	11.4±0.1	1.23±0.08
P6/2023-OSL6		108	100-200	228±17	4.9±0.2	11.5±1.3	11.3±0.8	1.14±0.18
P6-4		130	100-160	192±3	4.3±0.2	7.8±2.2	8.7±0.2	1.02±0.07
P6-5		150	100-150	230±015	8.6±0.4	17.8±2.3	18.0±1.0	0.98±0.06
P6/2023-OSL5		152	100-200	239±17	8.6±0.4	18.1±2.9	18.2±1.2	1.36±0.16
P6/2023-OSL4		182	100-200	211±21	8.2±0.4	15.3±1.9	15.9±1.4	1.21±0.08
P6-8		240	100-160	201±3	6.4±0.3	10.0±2.9	11.9±0.2	1.12±0.07
P6-12		270	100-150	201±3	7.4±0.4	13.1±2.8	13.2±0.2	1.13±0.08
P6-13		300	100-150	161±3	5.1±0.1	11.8±2.4	12.5±0.2	0.96±0.06
P6/2023-OSL3		335	100-200	233±17	4.5±0.2	9.0±1.2	9.1±0.7	1.18±0.15
P6/2023-OSL2		370	100-200	69±5	1.5±0.1	13.7±1.5	13.1±0.8	0.60±0.66
P6/2023-OSL1		455	100-200	181±14	6.1±0.3	10.2±1.5	10.7±0.8	1.73±0.16

Supplementary Table 6. D_e values and age data. N/n = number of aliquots measured/accepted for data analyses; OD = overdispersion; De values for different approaches to calculate an average: CAM = Central Age Model, MAM = Minimum Age Model, FMM = Finite Mixture Model; and corresponding ages.

Sample	N/n	OD	Model	D_e CAM (Gy)	D_e Model (Gy)	Age CAM (ka)	Age Model (ka)
BUHAIS -1	45/39	0.33	MAM	34.38±1.86	23.2±1.57	44±3	**30±2**
BUHAIS -3	48/48	0.33	FMM	108.15±5.35	88.15±5.97	150±10	**123±10**
BUHAIS -4	48/48	0.31	MAM	110.59±5.12	73.82±4.35	90±6	**59±5**
BUHAIS -5	51/49	0.63	MAM	32.01±2.88	11.55±1.02	39±4	**14±1**
BUHAIS -6	53/48	0.42	MAM	126.21±2.96	84.36±7.37	142±6	**95±9**
BUHAIS -7	46/45	0.17	MAM	108.95±3.30	97.31±5.62	140±7	**125±9**
BUHAIS -8	45/41	0.38	MAM	47.31±2.69	35.39±2.35	47±3	**35±3**
BUHAIS -9	40/39	0.38	MAM	39.01±2.40	33.60±1.34	48±4	**41±2**
BUHAIS -10	43/40	0.37	MAM	24.54±1.43	15.04±1.15	28±2	**17±2**
HWM-1	20/20	0.11	CAM	24.96±0.68	--	**17±1**	--
HWM-2	20/19	0.05	CAM	23.26±0.56	--	**16±1**	--
P6-1	11/11	0.40	MAM	1.71±0.11	1.57±0.07	1.3±0.2	**1.2±0.1**
P6-2	14/13	0.28	MAM	3.26±0.22	2.84±0.38	2.2±0.2	**1.9±0.3**
P6-3	50/49	0.42	MAM	5.65±0.37	4.84±0.43	4.6±0.4	**3.9±0.4**
P6/2023-OSL6	40/31	0.37	MAM	7.49±0.52	4.94±0.73	6.6±0.6	**4.3±0.7**
P6-4	14/13	0.20	CAM	6.83±0.40	--	**6.7±0.6**	--
P6-5	50/49	0.31	MAM	8.72±0.39	8.31±0.46	8.8±0.6	**8.5±0.7**
P6/2023-OSL5	24/21	0.25	MAM	11.68±0.69	10.76±1.19	8.6±0.8	**7.9±0.9**
P6/2023-OSL4	24/24	0.23	MAM	13.47±0.70	12.84±0.61	11±1	**11±1**
P6-8	11/11	0.20	CAM	9.29±0.41	--	**8.3±0.7**	--
P6-12	50/49	0.34	FMM	10.43±0.52	10.94±0.45	9.2±0.9	**9.7±0.8**
P6-13	24/24	0.17	CAM	29.12±1.09	--	**30±2**	--
P6/2023-OSL3	24/23	0.32	MAM	61.30±4.20	45.84±5.67	52±5	**39±5**
P6/2023-OSL2	24/23	0.30	MAM	40.19±2.61	35.69±3.59	67±5	**59±7**
P6/2023-OSL1	24/16	0.32	CAM	>262.51±22.36	--	**>152±14**	--

References

1 Armitage, S. J. *et al.* The Southern Route "Out of Africa": Evidence for an Early Expansion of Modern Humans into Arabia. *Science* **331**, 453–456 (2011).

2 Richter, D., Richter, A. & Dornich, K. Lexsyg smart — a luminescence detection system for dosimetry, material research and dating application. *Geochronometria* **42**, 202–209 (2015).

3 Murray, A. S. & Wintle, A. G. Luminescence dating of quartz using an improved single-aliquot regenerative-dose protocol. *Radiation Measurements* **32**, 57–73 (2000). https://doi.org/https://doi.org/10.1016/S1350-4487(99)00253-X

4 Wintle, A. G. & Murray, A. S. A review of quartz optically stimulated luminescence characteristics and their relevance in single-aliquot regeneration dating protocols. *Radiation Measurements* **41**, 369–391 (2006). https://doi.org/https://doi.org/10.1016/j.radmeas.2005.11.001

5 Dietze, M. *et al.* The abanico plot: Visualising chronometric data with individual standard errors. *Quaternary Geochronology* **31**, 12–18 (2016). https://doi.org/https://doi.org/10.1016/j.quageo.2015.09.003

6 Galbraith, R. F. & Roberts, R. G. Statistical aspects of equivalent dose and error calculation and display in OSL dating: An overview and some recommendations. *Quaternary Geochronology* **11**, 1–27 (2012). https://doi.org/https://doi.org/10.1016/j.quageo.2012.04.020

7 Bretzke, K. *et al.* Multiple phases of human occupation in Southeast Arabia between 210,000 and 120,000 years ago. *Scientific Reports* **12**, 1600 (2022). https://doi.org/10.1038/s41598-022-05617-w

8 Mueller, D. *et al.* Luminescence chronology of fluvial and aeolian deposits from the Emirate of Sharjah, UAE. *Quaternary Research* **112**, 111–127 (2023). https://doi.org/10.1017/qua.2022.51

9 Preusser, F., Degering, D., Fülling, A. & Miocic, J. Complex Dose Rate Calculations in Luminescence Dating of Lacustrine and Palustrine Sediments from Niederweningen, Northern Switzerland. *Geochronometria* **50**, 28–49 (2023). https://doi.org/10.2478/geochr-2023-0003

10 Kreutzer, S. *et al.* Software in the context of luminescence dating: status, concepts and suggestions exemplified by the R package 'Luminescence'. *Ancient TL* **35**, 1–11 (2017). https://doi.org/https://doi.org/10.26034/la.atl.2017.513
