# Supplementary material for: Evidence from Buhais Rockshelter for human settlement in Arabia between 60,000 and 16,000 years ago
Source: Nat Commun. 2026 Mar 23;17:2502. doi: 10.1038/s41467-026-70681-z (PMC13009211; doi:10.1038/s41467-026-70681-z)
Supplement: Supplementary file 2 — Reporting Summary [file 41467_2026_70681_MOESM2_ESM.pdf]

nature portfolio

Corresponding author(s): Knut Bretzke, Adrian G. Parker

Last updated by author(s): 19/02/26

Reporting Summary

Nature Portfolio wishes to improve the reproducibility of the work that we publish. This form provides structure for consistency and transparency in reporting. For further information on Nature Portfolio policies, see our Editorial Policies and the Editorial Policy Checklist.

Statistics

For all statistical analyses, confirm that the following items are present in the figure legend, table legend, main text, or Methods section.

n/a	Confirmed	
☐	☒	The exact sample size (*n*) for each experimental group/condition, given as a discrete number and unit of measurement
☒	☐	A statement on whether measurements were taken from distinct samples or whether the same sample was measured repeatedly
☒	☐	The statistical test(s) used AND whether they are one- or two-sided<br>*Only common tests should be described solely by name; describe more complex techniques in the Methods section.*
☒	☐	A description of all covariates tested
☒	☐	A description of any assumptions or corrections, such as tests of normality and adjustment for multiple comparisons
☐	☒	A full description of the statistical parameters including central tendency (e.g. means) or other basic estimates (e.g. regression coefficient) AND variation (e.g. standard deviation) or associated estimates of uncertainty (e.g. confidence intervals)
☒	☐	For null hypothesis testing, the test statistic (e.g. *F*, *t*, *r*) with confidence intervals, effect sizes, degrees of freedom and *P* value noted<br>*Give P values as exact values whenever suitable.*
☒	☐	For Bayesian analysis, information on the choice of priors and Markov chain Monte Carlo settings
☒	☐	For hierarchical and complex designs, identification of the appropriate level for tests and full reporting of outcomes
☒	☐	Estimates of effect sizes (e.g. Cohen's *d*, Pearson's *r*), indicating how they were calculated

Our web collection on statistics for biologists contains articles on many of the points above.

Software and code

Policy information about availability of computer code

Data collection	*Provide a description of all commercial, open source and custom code used to collect the data in this study, specifying the version used OR state that no software was used.*
Data analysis	Luminescence data analyses were performed using functions implemented in the R package 'Luminescence' 1.1.2 and ADELE software version 2017.

For manuscripts utilizing custom algorithms or software that are central to the research but not yet described in published literature, software must be made available to editors and reviewers. We strongly encourage code deposition in a community repository (e.g. GitHub). See the Nature Portfolio guidelines for submitting code & software for further information.

Data

Policy information about availability of data

All manuscripts must include a data availability statement. This statement should provide the following information, where applicable:

- Accession codes, unique identifiers, or web links for publicly available datasets
- A description of any restrictions on data availability
- For clinical datasets or third party data, please ensure that the statement adheres to our policy

All data used in this paper are provided in the main text and supplementary information. Archaeological samples used here are stored by the Sharjah Archaeology Authority (SAA). Requests for access should be addressed to SAA (info@saa.shj.ae).

Research involving human participants, their data, or biological material

Policy information about studies with human participants or human data. See also policy information about sex, gender (identity/presentation), and sexual orientation and race, ethnicity and racism.

Reporting on sex and gender	n.a.
Reporting on race, ethnicity, or other socially relevant groupings	n.a.
Population characteristics	n.a.
Recruitment	n.a.
Ethics oversight	n.a.

Note that full information on the approval of the study protocol must also be provided in the manuscript.

Field-specific reporting

Please select the one below that is the best fit for your research. If you are not sure, read the appropriate sections before making your selection.

☐ Life sciences ☐ Behavioural & social sciences ☒ Ecological, evolutionary & environmental sciences

For a reference copy of the document with all sections, see nature.com/documents/nr-reporting-summary-flat.pdf

Ecological, evolutionary & environmental sciences study design

All studies must disclose on these points even when the disclosure is negative.

Study description	Excavation of an archaeological sequence and sampling of sediment to provide palaeoenvironmental and chronological context for the recovered prehistoric cultural remains.
Research sample	Four stratigraphically distinct archaeological records consisting of lithic artifacts were analyzed. Nine sediment samples from the archaeological excavation were collected and studied to determine the chronological context of the material culture. Two sections from areas adjacent to the archaeological site were sampled for palaeoenvironmental information and their chronometric context.
Sampling strategy	Archaeological excavation followed modern standard procedures, including excavations following geological layers, piece plotting of archaeological finds and samples as well as systematic sieving. Chronological samples were taken from archaeological layers and layers in-between in the excavation. In the palaeoenvironmental sections, chronometric samples were taken from multiple horizons to cover the entire depth of the deposits.
Data collection	Archaeological data was collected through caliper measurements, pencil drawings on paper. Data was digitally stored using a computer. Palaeoenvironmental and OSL samples were collected from profiles of trenches using trowels and collecting the sample in plastic bags. See also method section and supplementary information for details.
Timing and spatial scale	Excavations were conducted in five seasons in 2017, 2019, 2020, 2023 and 2024. Sampling for chronometric analysis was conducted in the archaeological site in 2018, 2019 and 2022. Palaeoenvironmental sampling was conducted in parallel to this.
Data exclusions	No data was excluded from the analyses.
Reproducibility	Five seasons of excavation in the archaeological site produced reproduceable results.
Randomization	n.a.
Blinding	n.a.

Did the study involve field work? ☒ Yes ☐ No

Field work, collection and transport

Field conditions	Excavations and sampling in the field were conducted in February and March under weather conditions that were well suited for archaeological excvation and sediment sampling.
Location	FIeld work was conducted at site Buhais Rockshelter, Emirate of Sharjah, United Arab Emirates at 25.007° N, 55.790° E

Access & import/export	A proposal for archaeological excavations and palaeoenvironmental sampling has been submitted to the responsible authority (Shrajha Archaeology Authority [SAA]) in 2016. SAA granted permssion to conduct field work from 2017 onwards. All access, import and export was conducted following the rules and guiding by the Sharjah Archaeology Authority.
Disturbance	There were no relevant disturbances. Weather conditions allowed excavtions at all planned weeks in all seasons.

Reporting for specific materials, systems and methods

We require information from authors about some types of materials, experimental systems and methods used in many studies. Here, indicate whether each material, system or method listed is relevant to your study. If you are not sure if a list item applies to your research, read the appropriate section before selecting a response.

Materials & experimental systems

n/a	Involved in the study
☒	☐ Antibodies
☒	☐ Eukaryotic cell lines
☐	☒ Palaeontology and archaeology
☒	☐ Animals and other organisms
☒	☐ Clinical data
☒	☐ Dual use research of concern
☒	☐ Plants

Methods

n/a	Involved in the study
☒	☐ ChIP-seq
☒	☐ Flow cytometry
☒	☐ MRI-based neuroimaging

Palaeontology and Archaeology

Specimen provenance	Sharjah Archaeology Authority provided permission for archaeological excavations and analyses of archaeological material to KB. Permissions for export of geological samples was issued to KB and AGP. Archaeological, palaeoenvironmental and chronometric data has been collected at site Buhais Rockshelter (25.007° N, 55.790° E), Mleha P6 (25.129817°, 55.852051°) and Huweimi (25.423477°, 55.840119°). Export permissions were linked to excavation permission issued in years 2017, 2019, 2020, 2023 and 20204.
Specimen deposition	The archaeological collection is housed in Sharjah and managed by the Sharjah Archaeology Authority.
Dating methods	Dating results presented here are based on the optically stimulated luminescence method (OSL) conducted in the Frank Preusser lab at the University of Freiburg, Germany. The radiation dose accumulated by the quartz grains since deposition (De) was determined for small (1 mm) aliquots of quartz (ca. 50 grains), very close to a single-grain level. About 40-50 aliquots were measured per sample for archaeological layers, and ca. 20 for most palaeoenvironmental sections. Dose rates and ages were calculated using ADELEv2017 software, taking into account longitude (55.5°E), latitude (25.0°N), altitude (178 m), and sample depth below surface for cosmic dose rate.

☒ Tick this box to confirm that the raw and calibrated dates are available in the paper or in Supplementary Information.

Ethics oversight	This procject followed the rules and regulations established by the Sharjah Archaeology Authority, which also has monitored compliance.

Note that full information on the approval of the study protocol must also be provided in the manuscript.

Plants

Seed stocks	n.a.
Novel plant genotypes	n.a.
Authentication	n.a.
